# Tackling the Threat of Rabies Reintroduction in Europe

**DOI:** 10.3389/fvets.2020.613712

**Published:** 2021-01-15

**Authors:** Santiago Vega, Laura Lorenzo-Rebenaque, Clara Marin, Rosana Domingo, Fernando Fariñas

**Affiliations:** ^1^Facultad de Veterinaria, Instituto de Ciencias Biomédicas, Universidad Cardenal Herrera-CEU, CEU Universities, Alfara del Patriarca, Spain; ^2^Instituto de Inmunología Clínica y Enfermedades Infecciosas. Grupo One Health, Malaga, Spain

**Keywords:** rabies, *Lyssavirus*, One Health, zoonosis, dog, bat, Europe

## Abstract

Rabies is one of the oldest, most important zoonoses worldwide due to its extreme and inevitably lethal nature, causing one death every 9 min worldwide. Recent reports have demonstrated that the *Lyssavirus* continues more alive than ever, despite the control carried out against the virus throughout Europe. In this context, this work reviews the main immunological implications, transmission risk factors and current prevention measures for virus control in Europe, and especially in Spain.

## Introduction

Rabies is one of the oldest and most important zoonoses worldwide, due to its extreme and inevitably lethal outcomes (1, 2). Although the enzootic transmission of rabies is through Carnivora (dogs, jackals, wolves, etc.) and Chiroptera (bats), it can spill over to other mammalian species such as humans, who can end up developing the disease (2). Each year, rabies is estimated to be responsible for 59,000 human cases, mostly in Africa and Asia, and ~99% of human rabies cases are acquired after direct contact with dogs (3–5). The infection usually causes acute progressive encephalitis, and death eventually occurs if it is not treated before symptoms appear (1, 4). Although rabies remains one of the most feared and important threats to public health in the 21st century, it is considered one of the neglected diseases (2, 3).

## What Have We Learned from Rabies Disease?

### Rabies, a Long Etiological History

Rabies is caused by a group of neurotropic viruses of the genus *Lyssavirus*, belonging to the family *Rhabdoviridae* and order *Mononegavirales* (1, 6). This disease has been known since at least the 23rd century BC (Before Christ) in the Eshuma Code of Babylon (1). Moreover, the Greek ancient world called the disease “lyssa” (after the Greek goddess of madness, rage, and frenzy), due to the clinical signs it presented (6, 7). Since then, rabies has been present worldwide, except in Antarctica (4). The *Lyssavirus* contains a single-stranded RNA genome of negative sense, which encodes five structural proteins: nucleoprotein (N), phosphoprotein (P), matrix protein (M), glycoprotein (G) and the RNA-dependent RNA polymerase (L), in the order 3′-N-P-M-G-L-5′ 7 (6). Currently, the International Committee on Taxonomy of Viruses (ICTV) has delineated the genus into seventeen species, plus one related virus not yet taxonomically assessed, and segregated into three phylogroups (I, II, III-IV) (Table 1) (8, 9).

**Table 1:** Classification of the species of the genus *Lyssavirus* and geographical distribution.

**Phylogroup**	**Specie**	**Virus name**	**Distribution**
I	*Aravan lyssavirus*	Aravan virus	Central Asia
I	*Australian bat lyssavirus*	Australian bat lyssavirus	Australia
I	*Bokeloh bat lyssavirus*	Bokeloh bat lyssavirus	Europe
I	*Duvenhage lyssavirus*	Duvenhage virus	Southern Africa
I	*European bat 1 lyssavirus*	European bat 1 lyssavirus	Europe
I	*European bat 2 lyssavirus*	European bat 2 lyssavirus	Europe
	*Gannoruwa bat lyssavirus*	Gannoruwa bat lyssavirus	Asia
III-IV	*Ikoma lyssavirus*	Ikoma lyssavirus	Africa
I	*Irkut lyssavirus*	Irkut virus	East Siberia
I	*Khujand lyssavirus*	Khujand virus	Central Asia
II	*Lagos bat lyssavirus*	Lagos bat virus	Africa
III-IV	*Lleida bat lyssavirus*	Lleida bat lyssavirus	Europe (Spain)
II	*Mokola lyssavirus*	Mokola virus	Sub-Saharan Africa
I	*Rabies lyssavirus*	Rabies virus	Worldwide (except several islands)
II	*Shimoni bat lyssavirus*	Shimoni bat virus	East Africa
I	*Taiwan bat lyssavirus*	Taiwan bat lyssavirus	–
III-IV	*West Caucasian bat lyssavirus*	West Caucasian bat virus	Caucasian region
I	–	Kotalahti bat lyssavirus	–
Unclassified viruses	–	Taiwan bat lyssavirus	–

Fifteen of the seventeen lyssavirus species are hosted by bats (10). Indeed, bats are considered to be the ancestral hosts of lyssaviruses, and although the risk of human exposure is low, sporadic human rabies cases infected through a bat bite have been reported (10–13). Thus, from an epidemiological point of view, there are two epidemiological cycles: terrestrial rabies, maintained by domestic and wild carnivores, and rabies in chiropterans, where the virus is maintained in colonies of bats, both blood-sucking and insectivores or frugivores (14). Furthermore, it is difficult to differentiate the symptoms of the disease caused by any *Lyssavirus* species (10, 11).

### Rabies, a Hazardous Disease

The disease has a marked neurotropic character and its action on the nervous system gives rise to a characteristic manifestation of disease with excitatory signs, hallucinations and hydrophobia (furious rabies), or signs of generalised paralysis and coma (paralytic rabies), as a consequence of generally fatal encephalomyelitis (1, 4). As there is no clinical treatment, post-exposure prophylaxis has been demonstrated to be the most effective method to control and prevent human rabies cases worldwide (4). Based on the importance of prevention, the European Union has been focused on the need for the elimination of wildlife rabies in European countries by 2020 (15). Moreover, rabies control under a One Health approach is a top priority for the World Health Organisation (WHO), Food and Agriculture Organisation (FAO), World Organisation for Animal Health (OIE), and Global Alliance for Rabies Control (GARC). These agencies have set the goal of the elimination of dog-mediated human rabies by 2030 (4).

### Diagnostic Tools

Rabies diagnosis has been historically based on histopathological methods, such as cytoplasmic inclusion bodies detection (Negri bodies) (16, 17). At present, this methodology has been replaced by immunofluorescence. Also, when virus isolation has been necessary, rabies tissue culture infection or the mouse inoculation test has been used (16, 17). Currently, new techniques are being used for the diagnosis of the virus, such as flow cytometry (18), mass spectrometry (19), immunochromatographic tests and the polymerase chain reaction (PCR) (17).

## Historical Overview of Rabies in Europe

Since the earliest descriptions of the disease, the presence of the virus in dogs and wildlife has been reported in European countries (20). In dogs, rabies was endemic until the enforcement of regulations in the keeping of dogs and development of the vaccination progressively eliminated rabies in dogs during the 20th century (20, 21). Although Pasteur developed the earliest effective vaccine against rabies in dogs in 1885, the development and implementation of domestic animal vaccination was carried out from the 1920s (1). With the development of these prevention measures, rabies in dogs was eliminated from European countries, except for the European part of Turkey (22).

However, during the 1940s and thereafter, the disease emerged and became established in the red fox (*Vulpes Vulpes*) population between Russia and Poland, spreading southwards and westwards, and at its maximum extension in the early 1990s reached Southern France (2, 23). At this point, several European governments, with financial support from the European Union, rolled out oral rabies vaccination (**ORV**) campaigns in foxes, laying out the vaccines in baits, which managed to control vulpine rabies (22, 24). The development of ORV was a breakthrough for fox rabies control, and has resulted in the elimination of fox rabies from western Europe, with rapid progress also being made toward elimination in eastern Europe (22). Nevertheless, another rabies wildlife epidemiological cycle, maintained by the racoon dog (*Nyctereutes procyonides*), has been developed in the Baltic countries, many parts of eastern Europe, and Finland (25, 26). The racoon dog was introduced into the European part of Russia by the fur industry from eastern Asia in the first half of the 20th century, and has expanded throughout Europe (25, 26). The potential role of the raccoon dog in rabies transmission has been proven in rabies-free European areas, where the virus re-emerged due to this infected host (27). Therefore, the blueprint for eliminating wildlife-mediated rabies has also been extended to raccoon dogs (21).

Currently, most European countries are considered free of classical rabies, with confirmed rabies cases restricted to bats (1). The first evidence of infected European bat was reported in 1954 in Germany (28). From then on, the cases in bats were regarded as scientific curiosities, until 1985, when the intensive surveillance established after the first human case revealed more than a thousand bat rabies cases in Europe (28, 29).

Six lyssaviruses are reported to circulate in Europe: *European bat 1 lyssavirus* (EBLV-1), *European bat 2 lyssavirus* (EBLV-2), *Bokeloh bat lyssavirus* (BBLV), *West Caucasian bat lyssavirus* (WCBV), *Lleida bat lyssavirus* (LLEBV) and *Kotalahti bat lyssavirus* (KBLV) (30). Four of the viruses, EBLV-1, EBLV-2, BBLV, and KBLV, are associated with European bats of the family *Vespertilionidae* (30). Moreover, the other two lyssaviruses, WCBV and LLEBV, are linked to bats of the family *Miniopteridae* (30). Of these, only EBLV-1 and EBLV-2 have been reported to have caused rabies in humans, and more than 90% of the bat rabies cases have been associated with the bat species *Eptesicus serotinus (****E. serotinus****)* infected by EBLV-1 (12). The last European Union One Health 2018 Zoonoses Report confirmed bats as a reservoir for rabies in Europe, as it was present in 2% of the bats sampled (31). In this sense, taking samples from bats is the cornerstone to the knowledge of the dynamics of rabies within their hosts (32). To this end, guidelines on passive and active surveillance programmes were established by a European research consortium, Med-Vet-Net (33). Passive surveillance could include the investigation of sick or dead bats of all indigenous bat species, for testing lyssavirus infections using the detection methods. Moreover, active surveillance comprises the monitoring of free-living indigenous bats for the detection of viral RNA or virus-neutralising antibodies (33, 34). However, in Europe all bat species have been protected, which should be considered in the design and the undertaking of the surveillance initiatives (32). These studies showed that the principal ecological factors for viral persistence have been cross-species mixing and host migration patterns (10). Thus, the public health hazard of bat rabies must not be underestimated in Europe (31).

### The Border Between Europe and the African Continent: The Special Situation of Rabies in Spain

Spain is a country located in South-Western Europe on the Iberian Peninsula. At the southern extreme, it is separated from Morocco in Africa by the Strait of Gibraltar sea channel. The two continents are separated by just fourteen kilometres. On the north-eastern border, the Iberian Peninsula is linked to the rest of Europe through the Pyrenees mountain range, which forms a natural border between Spain and France. The Canary Islands at the Atlantic Ocean and the cities of Ceuta and Melilla in the Northern African coast are also part of Spain.

The first evidence of rabies in Spain was reported in the first century AD (*Anno Domini*), in Roman times (35). Over the centuries, attempts to control rabies had been developing through measures such as the control of the movement of dogs and cats in 1786, or washing of bite wounds with soap in an attempt to prevent the disease in 1863 (35). During the 20th century, Spain focused all its efforts on the elimination of rabies with an anti-rabies programme, until 1966, when rabies was eliminated in dogs, and the country was declared free of rabies for the first time (2, 36). The European vulpine rabies never crossed the Pyrenees due to the French ORV programmes (2, 35). However, in 1975, a new outbreak was declared in Malaga, in southern Spain, which might been related to the geographical proximity with Africa and the intense traffic of people, and the most accepted hypothesis was an introduction from Morocco (35). During the epidemic, the disease spread to 133 animals, 76 dogs, 30 cats, two foxes and six of other species with one human fatality (37). To avoid dog-mediated transmission, health measures were established, which included a dogs census, vaccination of susceptible animals, physical confinement and quarantine measures for suspect animals, control and reduction in the number of stray dogs, and the monitoring of dog bite wounds (35). The outbreak continued until 1978, when Spain was considered rabies-free in terrestrial carnivores (36). Nevertheless, every year, the autonomous cities of Ceuta and Melilla report rabies cases, mostly imported from Morocco (36). Infected animals have been also imported into mainland Spanish territories disregarding the official border controls and eventually spreading to other European countries (38).

Since the control of rabies in non-flying mammals, there has been increasing attention to lyssaviruses associated with European bats (39). The first case of rabies in bats was recorded in 1987 in Valencia (eastern Spain), and some sporadic cases in bats have been reported since then (9). A study carried out from 1992 to 2000 showed that EBLV-1, or even other lyssaviruses with cross-reactive antibodies, could have been circulating among bats of different species in several areas of Spain (40). *Lyssavirus* antibodies were detected in four bat species: *Myotis myotis* (*M. myotis*), *Miniopterus schreibersii* (*M. schreibersii*), *Tadarida teniotis* (*T. tenioti*) and *Rhinolophus ferrumequinum* (*R. ferrumequinum*) (40). Moreover, these findings raised the possibility of *M. schreibersii* as a dispersion vector of the disease in southern Europe due to its seasonal migrations, and the possible African origin of the EBLV-1 present in Spain due to the migrations of *T. teniotis* and *M. schreibersii* (40). During 1998–2003, a study of EBLV-1 viruses from *E. isabellinus* in southern Spain revealed the close communities in which EBLV-1 independently circulates, and yet distinct from other EBLV-1 strains circulating in the serotine bats (41). In addition, recent studies have demonstrated the geographical expansion of EBLV-1 in *E. serotinus* and *E. isabellinus* across Iberian Peninsula from Europe (12). In 2011, a new lyssavirus was confirmed in a *M. schreibersii* in the City of Lleida (north-eastern Spain), analysed as part of the rabies surveillance programme in Spain (42). The novel *Lyssavirus* was named LLEBV, and was classified into phylogroup III (42).

According to the previous premises, the emergence of sporadic human cases due to a bat or dog bite would be potentially feasible. Moreover, the dog is the main source responsible for a possible outbreak in Spain, as there is a high risk of importing an infected dog (36, 43). Spain has been officially free of rabies in non-flying mammals since 1978, although two imported cases of human rabies have been recorded in the last 30 years (13). Rabies surveillance data for Spain for the period of 1978–2020 reported 113 cases of rabies in dogs, of which 18 were stray dogs, and two both in cats and horses. However, these cases, except one, were limited to the cities of Ceuta and Melilla, which are located in North Africa (13). Likewise, rabies in wildlife was confirmed in 39 bats, as well as one fox in Ceuta, during this period (13). Thus, rabies remains a public health concern in Spain, and the main risk factors should be considered.

## Cross-immunity Between Different Lyssaviruses

A certain degree of cross-immunity between different lyssaviruses that can produce rabies has been described. Studies so far seem to show that such cross-immunity is possible between antigenic and genotypically “close” viruses, but not between more “distant” lyssaviruses belonging to different phylogroups (44). Thus, vaccination against classical canine rabies (phylogroup I) appears to confer protection against infection by lyssaviruses belonging to the same phylogroup, but not against European and African lyssaviruses belonging to phylogroups II and III.

New lyssaviruses are being discovered frequently, and although the risk is low, many of these viruses cannot be neutralised by antibodies from the traditional vaccine administered for the prevention of this disease. In view of the goal to end human deaths from dog-mediated rabies from the world by 2030, considering the elimination of dog-mediated rabies from a great part of Europe, consideration should be given to these other viruses that can produce rabies in humans and animals, for which the current vaccine is not protective. Therefore, some experts consider it essential to produce a universal vaccine that covers a broad spectrum of these lyssaviruses (45).

## Principal Risk Factors of the Disease in Europe

Rabies is widely distributed worldwide, with only a few countries considered free of the disease (1). Since 1977, for rabies information exchange in Europe, the WHO Collaborating Centre for Rabies Surveillance and Research, Friedrich Loeffler Institute in Wusterhausen, Germany, has developed the Rabies Bulletin Europe (46). To date, several European countries have become rabies-free of terrestrial rabies, but rabies remains present in others (38). European rabies risk is based on distinct epidemiological situations: illegal importation of animals, travel to endemic regions, differences in dog vaccination programmes and wildlife rabies.

### Illegal Importation of Animals

Although the successful elimination of dog-mediated rabies has been witnessed in the majority of European countries, it remains in certain European countries, as well as on the borders of Europe (47). In this sense, to ensure a sufficient level of safety in rabies, a European regulation establish strict measures on the movement of pet animals into a Member State from another Member State (48). However, illegal importation of pets through failure of border controls remains one of the main risks for rabies in Europe, leading to sporadic cases of rabies in free regions from endemic countries (13, 47, 49). From 2012 to 2020, five illegally imported dogs infected with rabies have been reported in France (2012, 2015, and 2020), Spain (2013) and the Netherlands (2013) from endemic areas (13, 36, 50, 51). These situations have occurred mainly because of importation of animals as pets from North Africa, disregarding the legislation on the movement of pet animals into Europe, and without disclosing the imported animals to the veterinary border control staff and customs officials (38, 43, 52, 53).

Spain presents geographical proximity and a commercial relationship with North Africa, with two cities in North Africa bordering with Morocco (Ceuta and Melilla), and a long-standing historical commercial traffic, fishing activities and labour migration between the Spanish-Moroccan border (54). Currently, rabies is endemic throughout Morocco, with human death reported yearly, remaining a serious public health problem (53). The domestic dog is considered the main reservoir and vector of the virus, and for this reason mass annual vaccination campaigns are conducted (53). However, the concept of responsible dog ownership does not feature in the Moroccan legislation, and vaccination coverage has not exceeded 6% of the total dog population (53). This situation affects Moroccan citizens, and the country has also become a source of rabies for neighbouring European states (43, 53). Every year, imported cases from Morocco are identified in Ceuta and Melilla (36), so compulsory and free of charge rabies vaccination programmes are carried out in these cities, in an attempt to reduce the risk (55). Imported cases pose a threat of rabies reintroduction into rabies-free areas, highlighting the need for reinforcement of border surveillance (38, 56).

### Travel to Endemic Regions

Although the risk of a traveller contracting rabies is considered low, no prophylaxis or specific rabies vaccinations are needed for travel to endemic areas (52, 56). In 2019, four imported human rabies case were reported in Latvia, Spain, Italy, and Norway after having contracted the disease through a dog or cat bite while travelling in endemic areas (India, Morocco, Tanzania, and Philippines, respectively) (57). The imported cases of human rabies reflect lack of awareness by travellers visiting rabies-endemic countries, especially in Africa and Asia (43, 52, 57). Travellers to endemic areas should be aware of this, acting with caution and avoiding touching all animals, including puppies, to prevent animal bites (9, 57, 58). Moreover, the recommendations include local wound care, the vaccination, and if indicated, passive immunisation (9).

In addition, the risk increases when travelling with animals. In this sense, recent reports related lack of awareness by travellers of the risk posed by taking their non-vaccinated dogs abroad to an endemic region, or by adopting animals from these areas and taking them back home with them (43, 47).

### Differences in Dog Vaccination Programmes

As there is no clinical treatment for this zoonotic disease, and dogs represent a main source of human infection, prevention by vaccination is the mainstay approach to avoid the spread of rabies (1). According to the WHO, vaccination coverage should reach 70% of the dog population to prevent rabies transmission (4). Regular application of the vaccines provides a more cost-effective basic instrument than the post-bite treatment of rabies cases, both short-term and longer-term (1). Indeed, the cost of a post-bite treatment in humans is around US$ 100, while dog vaccination costs are around US$ 0.50 per dog (59).

The WHO Expert Committee on Rabies considered mass vaccination programmes the basis of canine rabies control (46). Moreover, it was recommended that these mass vaccination programmes, which included the primary immunisation of all dogs between 3 months and 1 year old, should be carried out annually, and also emphasised the importance of including cats in these programmes (46).

The threat of rabies requires a constant state of alert, as an immunisation rate of <70% poses a risk to herd immunity in Europe (60). Furthermore, the geographical proximity to territories that are not free of rabies should be of special consideration, as it will pose a risk to the whole of Europe.

In addition to the lack of a coordinated vaccination programme, vaccine failures may occur, due to causes such as failure to administer the vaccine, poor quality rabies vaccine, and poor immune, health and nutritional status of the vaccinated animal (46). In addition, a single shot of vaccine is not enough to achieve long-lasting optimal immunity against rabies, leading to insecure rabies protection rates in a biting animal, despite a history of rabies vaccination (1).

### Circulation of the Virus Among Wildlife population

Insectivorous bat species have often been employed as a mosquito biocontrol strategy, and it has been observed that a single bat is able to consume up to 600 mosquitoes per hour (61). Mosquitoes may serve as vectors of mosquito-borne diseases such as Zika virus, West Nile virus, malaria, or dengue (62). This highlights the important role of insectivorous bats in the reduction of mosquito populations and their implication in the protection against mosquito-related disease (63). In fact, in Spain bats are used as a control measure for mosquito populations, although some of the species used have been found positive for the presence of lyssaviruses (30, 64). Even though the risk of possible transmission of bat lyssaviruses to terrestrial mammals are very low in European countries, human and animal rabies cases following a bat bite have been reported (13, 65). Besides, exposure to bats should be regarded as a potential rabies risk in Europe, especially for spelunkers or bat biologists, who have a high risk of contact with rabid bats (38, 66). Recently, Italy has reported a case of WCBV in a cat, which lived near to bat colonies, that could represent the source of the virus (67). Ecological factors such as cross-species mixing and migrations of bat populations could be responsible for the viral persistence in European bats (10).

In Europe, the chiropteran rabies cycle is independent from the terrestrial rabies cycle (24). In 2019, the surveillance data confirmed five cases in animals in Europe, the highest proportion of the cases being in red foxes (two in Romania and one in Poland), followed by one case in a cow and in a wild boar, also in Romania (68). In the early 1960s, red fox rabies emerged in many European countries (13, 25). Over the past three decades, European fox-mediated rabies has been successfully controlled and eliminated in response to the effective implementation of the ORV programmes (13, 69). Countries can be officially declared free of terrestrial rabies when no cases have been detected for a 2-year period (15), a status achieved by European countries such as Finland and The Netherlands in 1991, Italy in 1997, Switzerland in 1998, France in 2000, Belgium and Luxembourg in 2001, the Czech Republic in 2004 and Germany and Austria in 2008 (13). Despite the success achieved, in Italy fox rabies re-emerged in 2009 (13), and a recent emergence and spread of fox rabies into previously rabies-free areas, due to insufficient cross-border cooperation and a false sense of security, highlights the need to stay alert (15, 49).

## Will Rabies Ever Be Prevented in Europe?

Although rabies is 100% preventable, one person dies from it every 9 min worldwide (4). Although most of the cases are in Africa and Asia, the public health hazard of rabies must not be underestimated in Europe, because of all the risks their countries present (4, 31). Rabies prevention is based on three cornerstones: (1) improving education and public awareness; (2) access to mass dog vaccination; and (3) increasing the access to treatment medicines and vaccines (4).

### Improving Education and Public Awareness

Despite 3 billion people continuing to be at risk of rabies worldwide, rabies continues to be a largely neglected disease (4, 70). In this sense, the European population often overstates the health security of rabies and surveillance should continue (71). Awareness and understanding of how to prevent the disease in animals, when there are reasons to suspect rabies, and what to do in the event of a bite, are crucial to save people (4), not only in endemic areas, but also in rabies-free countries.

In Europe, legislative measures for the control of this zoonosis have been implemented (72). Nevertheless, stricter laws should be enforced to raise awareness of the potential risks of rabies disease and prevent the introduction of rabies into rabies-free European countries (1, 73). These measures should be focused on raising awareness mainly on travel to endemic areas, travelling with pets, and importing and trading in animals from endemic areas (1).

### Access to Mass Dog Vaccination

Vaccination of dogs is the key to curtailing rabies transmission between dogs, and from dogs to humans. For ethical, ecological and economic reasons, the slaughtering of rabies vectors should not be considered as a main control and eradication method (74). Prevention of the transmission of rabies at its source through dog vaccination is the most cost-effective strategy to save lives (4). Although rabies control programmes involve a high cost in many countries, the cost of pre-exposure dog vaccination is much lower than the current cost of the emergency post-exposure treatment (74). In fact, just 10% of the financial resources used in post-exposure treatment would able to significantly reduce, or even eliminate, the disease in the canine population, and consequently, the number of human cases (74).

In wildlife, ORV baits have proven to be an effective sophisticated strategy to control and even eliminate fox rabies in European countries, mitigating rabies risks to humans (75). Nevertheless, few eastern European countries and countries bordering Europe are still trying to control it, posing a risk to free-rabies wildlife countries (22). For this reason, the European oral vaccination financing programmes continue to be a necessary strategy, as they are the basis of rabies control in wildlife (22).

### Increased Access to Treatment Medicines and Vaccines

The best method of rabies prevention is to avoid bites from mammals, especially high-risk rabies reservoir species (1). However, this is difficult to achieve, as accidental exposures are very common (1). Worldwide, post-bite vaccination incidence has been estimated at 29 million people in a year (9). Appropriate wound management and immediate and adequate post-exposure treatment is almost 100% effective in preventing human rabies deaths (4).

In any case, the severity of the process will mainly depend on the place and type of bite and the speed of the post-exposure prophylaxis (76). Therefore, the importance of knowing the risk to which the population are subjected and the ability to react is the basis for being able to save lives, and because of this, rabies is the only disease where there is a post-exposure immunoprophylaxis protocol in humans (77). Treatment consists of thoroughly washing the bite wound with soap, and if possible with viricidal antiseptic (e.g., povidone iodine or ethanol) for at least 15 min, followed by the administration of passive immunisation with immunoglobulins, and vaccination for active immunity (46).

## Final Comments and Future Directions

The risk of importing dog rabies cases from North Africa is in increasingly important evidence. Given this situation, the health authorities must increase surveillance, especially at the entry points to the Iberian Peninsula, as motor vehicles entering can illegally transport animals that are sick or in incubation period from North Africa.

Although the risk of importing rabies cases from other territories within Europe is considered to be lower than in the case of North Africa, it should be considered that (1) the free movement of persons and goods in EU countries easily allows the entry of illegally-transported animals from countries that present cases of rabies in domestic animals (mainly dog and cat), and even in wild species (mainly red fox); (2) the lack of specific measures and some relaxation of controls concerning vulpine populations may facilitate outbreaks in countries declared free of this type of rabies; (3) it should not be forgotten that the spread of rabies from sick foxes to dogs is well established, and *vice versa*, as seems to have been in the case of Spain in 1977, when two cases of rabies in foxes were described in the province of Malaga during the 1975 rabies outbreak. These were fortunately isolated cases, unrelated to each other, probably due to the single infection from sick dogs or the consumption of carrion from carcasses.

It is considered necessary to maintain a single criterion throughout Europe regarding compulsory vaccination, which must be annual (depending on the authorised product used for immunisation, which must be applied by authorised veterinarians) to guarantee sufficient protective immunity against rabies, in any case systematically, and which must cover all dogs and cats without exception, as in the case of ferrets or raccoon dogs, which are also particularly susceptible to the rabies virus.

Given the recent cases of bat rabies described in Europe and the considerations set out above concerning the possibility of transmission to terrestrial species, as well as the arrival of animals from other latitudes, whatever the cause, a continuous state of alert of the competent authorities is required. In the same vein, the authorities responsible should encourage and provide adequate funding for specialised research groups to constantly improve the resources available for diagnosis and vaccination, and to search for new vaccine products capable of protecting humans and animal populations from possible exposure to these viruses, which sometimes have insufficient links with classical rabies virus to ensure adequate protection.

## Author Contributions

All authors wrote the manuscript and performed all the necessary literature searches and data compilation. All authors approved the submitted version.

## Conflict of Interest

The authors declare that the research was conducted in the absence of any commercial or financial relationships that could be construed as a potential conflict of interest.

## References

[1] Garg SR Rabies in Man and Animals. 1st ed Haryana: Springer (2014).

[2] Mingo-Casas P Sandonis V Vázquez-Morón S Juste J Rabies in Spain. A peculiarity in Eurasia The impact on linkage-to-care of an alternative hepatitis C screening method in PWID view project. Ann Virol Res. (2017) 3:1030.

[3] Fooks AR Banyard AC Horton DL Johnson N Mcelhinney LM Jackson AC. Current status of rabies and prospects for elimination. Lancet. (2014) 384:1389. 10.1016/S0140-6736(13)62707-5 24828901 PMC7159301

[4] WHO-FAO-OIE WHO | Zero by 30: The Global Strategic Plan to End Human Deaths From Dog-Mediated Rabies by 2030. (2018). Available online at: http://www.who.int/rabies/resources/9789241513838/en/ (accessed May 28, 2020).

[5] Fisher CR Streicker DG Schnell MJ. The spread and evolution of rabies virus: conquering new frontiers. Nat Rev Microbiol. (2019) 16:241–55. 10.1038/nrmicro.2018.11 29479072 PMC6899062

[6] ICTV. Genus: Lyssavirus - Rhabdoviridae - Mononegavirales - International Committee on Taxonomy of Viruses (ICTV). (2019). Available online at: https://talk.ictvonline.org/ictv-reports/ictv_online_report/negative-sense-rna-viruses/mononegavirales/w/rhabdoviridae/795/genus-lyssavirus (accessed May 28, 2020).

[7] Neville J Rabies in the Ancient World. In: King A Fooks AR Aubert M Wandeler AI, editors. Historical Perspective of Rabies in Europe and the Mediterranean Basin. Paris: OIE (2004). p. 1–12.

[8] Shipley R Wright E Selden D Wu G Aegerter J Fooks AR. Bats and viruses: emergence of novel lyssaviruses and association of bats with viral zoonoses in the EU. Trop Med Infect Dis. (2019) 4:31. 10.3390/tropicalmed4010031 30736432 PMC6473451

[9] WHO Classification | Rabies - Bulletin – Europe. (2020). Available online at: https://www.who-rabies-bulletin.org/site-page/classification (accessed November 2, 2020).

[10] Colombi D Serra-Cobo J Métras R Apolloni A Poletto C López-Roig M. Mechanisms for lyssavirus persistence in non-synanthropic bats in Europe: insights from a modeling study. Sci Rep. (2019) 9:1–11. 10.1038/s41598-018-36485-y 30679459 PMC6345892

[11] Scheffer C Asano M Garcia E Samira M Fahl DO. Murciélagos hematófagos como reservorios de la rabia. Rev Peru Med Exp Salud Publica. (2014) 31:302–9. 10.17843/rpmesp.2014.312.51 25123871

[12] Mingo-Casas P Sandonís V Obón E Berciano JM Vázquez-Morón S Juste J. First cases of European bat lyssavirus type 1 in Iberian serotine bats: implications for the molecular epidemiology of bat rabies in Europe. PLoS Negl Trop Dis. (2018) 12:1–9. 10.1371/journal.pntd.0006290 29684025 PMC5933805

[13] WHO Rabies. (2020). Available online at: https://www.who.int/news-room/fact-sheets/detail/rabies (accessed May 28, 2020).

[14] Cifuentes JF Pérez RD Verjan N Bat reservoirs for rabies virus and epidemiology of rabies in Colombia: a review. CES Med Vet Zootec. (2017) 12:134–50. 10.21615/cesmvz.12.2.5

[15] European Commission DG Health and Food Safety Overview Report - Rabies Eradication in the EU. Luxembourg (2017).

[16] OIE Manual of Diagnostic Tests and Vaccines for Terrestrial Animals. 6th ed Vol 1 Paris: OIE (2008).

[17] Rupprecht CE Fooks AR Abela-Ridder B Laboratory Techniques in Rabies. 5th ed Vol 1 Geneva: World Health Organization (2018).

[18] Bordignon J Pires Ferreira SC Medeiros Caporale GM Carrieri ML Kotait I Lima HC. Flow cytometry assay for intracellular rabies virus detection. J Virol Methods. (2002) 105:181–6. 10.1016/S0166-0934(02)00064-2 12176155

[19] Reed M Stuchlik O Carson WC Orciari L Yager PA Olson V. Novel mass spectrometry based detection and identification of variants of rabies virus nucleoprotein in infected brain tissues. PLoS Negl Trop Dis. (2018) 12:e0006984. 10.1371/journal.pntd.0006984 30550539 PMC6310296

[20] Blancou J Rabies in Europe and the Mediterranean basin: from antiquity to the 19th Century. In: eds. King A Fooks AR Aubert M Wandeler AI, editors. Historical Perspective of Rabies in Europe and the Mediterranean Basin. Paris: OIE (2004). p. 15–23.

[21] Müller FT Freuling CM. Rabies control in Europe: an overview of past, current and future strategies. Rev Sci Tech. (2018) 37:409–19. 10.20506/rst.37.2.2811 30747138

[22] Freuling CM Hampson K Selhorst T Schröder R Meslin FX Mettenleiter TC. The elimination of fox rabies from Europe: Determinants of success and lessons for the future. Philos Trans R Soc B Biol Sci. (2013) 368:20120142. 10.1098/rstb.2012.0142 23798690 PMC3720040

[23] Westerling B Andersons Z Rimeicans J Lukauskas K Dranseika A Rabies in the baltics. In: King A Fooks AR Aubert M Wandeler AI, editors. Historical Perspective of Rabies in Europe and the Mediterranean Basin. París: OIE (2004). p. 33–44.

[24] European Commission The Oral Vaccination of Foxes Against Rabies Report of the Scientific Committee on Animal Health and Animal Welfare (2002).

[25] Mähl P Cliquet F Guiot AL Niin E Fournials E Saint-Jean N. Twenty-year experience of the oral rabies vaccine SAG2 in wildlife: A global review. Vet Res. (2014) 45:1–17. 10.1186/s13567-014-0077-8 25106552 PMC4423639

[26] Robardet E Picard-Meyer E Dobroštana M Jaceviciene I Mähar K MuiŽniece Z. Rabies in the Baltic States: decoding a process of control and elimination. PLoS Negl Trop Dis. (2016) 10:1–26. 10.1371/journal.pntd.0004432 26849358 PMC4743931

[27] Sutor A Schwarz S Conraths FJ. The biological potential of the raccoon dog (Nyctereutes procyonoides, Gray 1834) as an invasive species in Europe-new risks for disease spread? Acta Theriol. (2014) 59:49–59. 10.1007/s13364-013-0138-9 32226062 PMC7097217

[28] Müller W Cox J Müller T. Rabies in Germany, Denmark and Austria. In: King A Fooks AR Aubert M Wandeler AI, editors. Historical Perspective of Rabies in Europe and the Mediterranean Basin. Paris: OIE (2004).

[29] Picard-Meyer E Robardet E Arthur L Larcher G Harbusch C Servat A. Bat rabies in France: A 24-year retrospective epidemiological study. PLoS ONE. (2014) 9:e98622. 10.1371/journal.pone.0098622 24892287 PMC4044004

[30] Banyard AC Evans JS Luo TR Fooks AR. Lyssaviruses and bats: Emergence and zoonotic threat. Viruses. (2014) 6:2974–90. 10.3390/v6082974 25093425 PMC4147683

[31] EFSA. The European Union One Health 2018 zoonoses report. EFSA J. (2019) 17:17. 10.2903/j.efsa.2019.5926 32626211 PMC7055727

[32] Leopardi S Priori P Zecchin B Poglayen G Trevisiol K Lelli D. Active and passive surveillance for bat lyssaviruses in Italy revealed serological evidence for their circulation in three bat species. Epidemiol Infect. (2019) 147:1–6. 10.1017/S0950268818003072 30511606 PMC6518613

[33] Schatz J Fooks AR Mcelhinney L Horton D Echevarria J Vázquez-Moron S. Bat rabies surveillance in Europe. Zoonoses Public Health. (2013) 60:22–34. 10.1111/zph.12002 22963584

[34] Schatz J Freuling CM Auer E Goharriz H Harbusch C Johnson N. Enhanced passive bat rabies surveillance in indigenous bat species from Germany - a retrospective study. PLoS Negl Trop Dis. (2014) 8:2835. 10.1371/journal.pntd.0002835 24784117 PMC4006713

[35] Abellan-Garcia C Sánchez-Serrano LP Amador R Rosinha AJ Rabies in the Iberian Peninsula. In: King A Fooks AR Aubert M Wandeler AI, editors. Historical Perspective of Rabies in Europe and the Mediterranean Basin. Paris: OIE (2004). p. 147–55.

[36] Pérez de Diego AC Vigo M Monsalve J Escudero A. The One Health approach for the management of an imported case of rabies in mainland Spain in 2013. Eurosurveillance. (2015) 20:1–5. 10.2807/1560-7917.ES2015.20.6.21033 25695478

[37] Rodríguez-Ferri E Estado actual de la rabia animal, con especial referencia a España. Colección Vet Salud Públ Min Sanidad Consumo. (1987) 4:1–204.

[38] Cliquet F Picard-Meyer E Robardet E. Rabies in Europe: what are the risks? Expert Rev Anti Infect Ther. (2014) 12:905–8. 10.1586/14787210.2014.921570 24847903

[39] King AA Haagsma J Kappeler A Lyssavirus infections in European bats In: King AA Fooks AR Aubert M Wandeler AI, editors. Historical Perspective of Rabies in Europe and the Mediterranean Basin. Paris: OIE (2004).

[40] Serra-Cobo J Amengual B Carlos Abellán B Bourhy H. European bat Lyssavirus infection in Spanish bat populations. Emerg Infect Dis. (2002) 8:413–20. 10.3201/eid0804.010263 11971777 PMC2730232

[41] Vázquez-Morón S Juste J Ibáñez C Ruiz-Villamor E Avellón A Vera M. Endemic circulation of European bat lyssavirus type 1 in serotine bats, Spain. Emerg Infect Dis. (2008) 14:1263–6. 10.3201/1408.080068 18680651 PMC2600403

[42] Aréchiga-Ceballos N Morón SV Berciano JM Nicolás O López CA Juste J. Novel lyssavirus in bat, Spain. Emerg Infect Dis. (2013) 19:793–5. 10.3201/eid1905.121071 23648051 PMC3647500

[43] Napp S Casas M Moset S Paramio JL Casal J. Quantitative risk assessment model of canine rabies introduction: Application to the risk to the European Union from Morocco. Epidemiol Infect. (2010) 138:1569–80. 10.1017/S0950268810000415 20199698

[44] Echevarría JE Banyard AC McElhinney LM Fooks AR. Current rabies vaccines do not confer protective immunity against divergent lyssaviruses circulating in Europe. Viruses. (2019) 11:892. 10.3390/v11100892 31554170 PMC6832729

[45] Evans JS Horton DL Easton AJ Fooks AR Banyard AC. Rabies virus vaccines: Is there a need for a pan-lyssavirus vaccine? Vaccine. (2012) 30:7447–54. 10.1016/j.vaccine.2012.10.015 23084854

[46] WHO WHO Technical Report Series 931 WHO Expert Consultation on Rabies First Report (2005). 16485446

[47] Bourhy H Dacheux L Strady C Mailles A Rabies in Europe in 2005. Euro Surveill. (2005) 10:213–6. 10.2807/esm.10.11.00575-en 16371690

[48] European Parliament and the Council of the European Union Regulation (EC) No. 576/2013 of the European Parliament and of the Council of 12 June 2013 on the Non-commercial Movement of Pet Animals and Repealing Regulation (EC) No 998/2003. J Eur Union L. (2013) 178:1−26.

[49] Müller T Freuling CM Wysocki P Roumiantzeff M Freney J Mettenleiter TC. Terrestrial rabies control in the European Union: Historical achievements and challenges ahead. Vet J. (2015) 203:10–7. 10.1016/j.tvjl.2014.10.026 25466578

[50] Berger S Rabies: Global Status. 2018th ed Los Angeles, CA: GIDEON Informatics Inc. (2018).

[51] ECDC Rabies Annual Epidemiological Report for 2018. Stockholm (2018). p. 2000–2.

[52] Gautret P Ribadeau-Dumas F Parola P Brouqui P Bourhy H. Risk for rabies importation from North Africa. Emerg Infect Dis. (2011) 17:2187–93. 10.3201/eid1712.110300 22185767 PMC3311213

[53] Darkaoui S Cliquet F Wasniewski M Robardet E Aboulfidaa N Bouslikhane M. A century spent combating rabies in Morocco (1911-2015): How much longer? Front Vet Sci. (2017) 4:78. 10.3389/fvets.2017.00078 28626749 PMC5454081

[54] Ferrer-Gallardo X The Spanish-Moroccan border complex: Processes of geopolitical, functional and symbolic rebordering. Polit Geogr. (2008) 27:301–21. 10.1016/j.polgeo.2007.12.004

[55] WHO WHO Rabies Bulletin Europe, No 2. (2013). p. 1–24. Available online at: http://www.who-rabies-bulletin.org/ (accessed May 29, 2020)

[56] Ribadeau-Dumas F Cliquet F Gautret P Robardet E Le Pen C Bourhy H. Travel-associated rabies in pets and residual rabies risk, Western Europe. Emerg Infect Dis. (2016) 22:1268–71. 10.3201/eid2207.151733 27314463 PMC4918150

[57] ECDC Fourth Travel-Related Rabies Case Reported in the EU in 2019. (2019). Available online at: https://www.ecdc.europa.eu/en/news-events/fourth-travel-related-rabies-case-reported-eu-2019 (accessed June 3, 2020).

[58] Malerczyk C DeTora L Gniel D. Imported human rabies cases in Europe, the United States, and Japan, 1990 to 2010. J Travel Med. (2011) 18:402–7. 10.1111/j.1708-8305.2011.00557.x 22017716

[59] WHO WHO | Vaccinate Dogs to Save Human Lives – World Rabies Day 2012. WHO (2012).

[60] Hampson K Dushoff J Cleaveland S Haydon DT Kaare M Packer C. Transmission dynamics and prospects for the elimination of canine rabies. PLoS Biol. (2009) 7:e1000053. 10.1371/journal.pbio.1000053 19278295 PMC2653555

[61] Gonsalves L Bicknell B Law B Webb C Monamy V. Mosquito consumption by insectivorous bats: does size matter? PLoS ONE. (2013) 8:1–11. 10.1371/journal.pone.0077183 24130851 PMC3795000

[62] Zeller H Marrama L Sudre B Van Bortel W Warns-Petit E Mosquito-borne disease surveillance by the European Centre for Disease Prevention and Control. Clin Microbiol Infect. (2013) 19:693–8. 10.1111/1469-0691.12230 23607415

[63] Reiskind MH Wund MA. Bats & Mosquitoes Testing Conventional Wisdom. Texas: Bat Conservation International. (2010).

[64] UCM Universidad Complutense de Madrid. Texas: Bat Conser vation International (2016). Available at: https://www.ucm.es/el-grupo-de-seguimiento-de-fauna-cei-campus-moncloa-aboga-por-el-uso-de-murcielagos-como-control-de-plagas-en-casos-como-el-mosquito-que-trasmite-el-virus-zika-24-de-febrero (accessed June 2, 2020).

[65] Racey PA Hutson AM Lina PHC. Bat rabies, public health and European bat conservation. Zoonoses Public Health. (2013) 60:58–68. 10.1111/j.1863-2378.2012.01533.x 22909028

[66] Krzowska-Firych J Tomasiewicz K Kozøowska A. Post-exposure rabies prophylaxis in humans exposed to animals in Lublin province (Eastern Poland) in 2012–2015–A retrospective study. Hum Vaccines Immunother. (2017) 13:1346–51. 10.1080/21645515.2017.1285474 28166482 PMC5489294

[67] Coxon C McElhinney L Pacey A Gauntlett F Holland S Preliminary Outbreak Assessment: Rabies in a Cat in Italy. (2020). Available online at: https://www.who-rabies-bulletin.org/site-page/classification (accessed November 10, 2020).

[68] Gossner CM Mailles A Aznar I Dimina E Echevarría JE Feruglio SL. Prevention of human rabies: a challenge for the European Union and the European Economic Area. Eurosurveillance. (2020) 25:2000158. 10.2807/1560-7917.ES.2020.25.38.2000158 32975184 PMC7533618

[69] Müller T Bätza HJ Freuling C Kliemt A Kliemt J Heuser R Elimination of terrestrial rabies in Germany using oral vaccination of foxes. Berl Munch Tierarztl Wochenschr. (2012) 125:178–90. 22712414

[70] Wunner WH Briggs DJ Rabies in the 21st century. PLoS Negl Trop Dis. (2010) 4:591 10.1371/journal.pntd.0000591 PMC2846934 20361028

[71] Finnegan CJ Brookes SM Johnson N Smith J Mansfield KL Keene VL. Rabies in North America and Europe. J R Soc Med. (2002) 95:9–13. 10.1177/014107680209500104 11773344 PMC1279140

[72] Directive 2003/99/EC Directive 2003/99/EC of the European Parliament and of the Council of 17 November 2003 on the Monitoring of Zoonoses and Zoonotic Agents, Amending Council Decision 90/424/EEC and Repealing Council Directive 92/117/EEC (2003).

[73] Lembo T Partners for Rabies Prevention. The Blueprint for Rabies Prevention and Control: A Novel Operational Toolkit for Rabies Elimination. PLoS Negl Trop Dis. (2012) 6:e1388. 10.1371/journal.pntd.0001388 22389727 PMC3289591

[74] OIE OIE's Commitment to Fight Rabies Worldwide. (2014). Available online at: https://www.oie.int/doc/ged/D14107.PDF (accessed November 14, 2020).

[75] Maki J Guiot AL Aubert M Brochier B Cliquet F Hanlon CA. Oral vaccination of wildlife using a vaccinia-rabies-glycoprotein recombinant virus vaccine (RABORAL V-RG®): A global review. Vet Res. (2017) 48:57. 10.1186/s13567-017-0459-9 28938920 PMC5610451

[76] Andrade BF Andrade TS Queiroz LH. Human rabies post-exposure prophylaxis relative to the disease epidemiological status. Cienc Saude Coletiva. (2019) 24:315–22. 10.1590/1413-81232018241.32832016 30698264

[77] Briggs DJ. The role of vaccination in rabies prevention. Curr Opin Virol. (2012) 2:309–14. 10.1016/j.coviro.2012.03.007 22503445

